# Life‐course socioeconomic position and mild cognitive impairment in U.S Hispanic/Latino adults: Results from the HCHS/SOL and SOL‐INCA

**DOI:** 10.1002/alz70860_105160

**Published:** 2025-12-23

**Authors:** Paola Filigrana, Linda C Gallo, Myriam Fornage, Charles Decarli, Jee‐Young Moon, Krista M Perreira, Humberto Parada, Jianwen Cai, Frank J Penedo, Maria M Llabre, Amber Pirzada, Martha L Daviglus, Wassim Tarraf, Freddie Márquez, Shakira F Suglia, Hector M Gonzalez, Carmen R Isasi

**Affiliations:** ^1^ Albert Einstein College of Medicine, Bronx, NY, USA; ^2^ San Diego State University, San Diego, CA, USA; ^3^ Human Genetics Center, School of Public Health, University of Texas Health Science Center, Houston, TX, USA; ^4^ The Brown Foundation Institute of Molecular Medicine, McGovern Medical School, The University of Texas Health Science Center at Houston, Houston, TX, USA; ^5^ University of Texas Health Science Center at Houston, Houston, TX, USA; ^6^ University of California, Davis, Davis, CA, USA; ^7^ IDeA Laboratory, Department of Neurology, UC Davis, Davis, CA, USA; ^8^ University of North Carolina, Chapel Hill, NC, USA; ^9^ University of Miami, Coral Gables, FL, USA; ^10^ University of Miami, Miami, FL, USA; ^11^ University of Illinois, Chicago, IL, USA; ^12^ University of Illinois at Chicago, Chicago, IL, USA; ^13^ Wayne State University, Detroit, MI, USA; ^14^ University of California, San Diego, La Jolla, CA, USA; ^15^ Emory University, Atlanta, GA, USA

## Abstract

**Background:**

The burden of Alzheimer's Disease and Related Dementias (ADRD) is high in US Hispanic/Latino adults. Cumulative exposure to socioeconomic disadvantage over the life‐course may help contribute to this high ADRD burden. We examined the association between socioeconomic position (SEP) over the life‐course with mild cognitive impairment (MCI) in middle‐aged and older Hispanic/Latino adults.

**Methods:**

We used data from the Hispanic Community Health Study/Study of Latinos (HCHS/SOL), a multicenter community‐based cohort of US Hispanic/Latino adults (baseline 2008‐2011) and the SOL‐INCA ancillary study conducted ∼7 years later (2015‐2018) (*n* = 6351; age 50‐86 years). Childhood SEP was determined using parental education (<high school [HS] or ≥HS). Adult SEP at baseline was operationalized using an index combining participants’ education, household income, employment, and assets (range 0‐7) and categorized at the median as low and high SEP. Using childhood and adult SEP, we classified participants into four socioeconomic mobility categories: stable low SEP (*n* = 1539); upward mobility (*n* = 2040); downward mobility (*n* = 426); or stable high SEP (*n* = 1223). MCI (yes or no) was defined in the follow‐up visit using NIA‐Alzheimer's Association diagnostic criteria (i.e., any cognitive score in the mildly impaired range, significant cognitive decline, self‐reported cognitive decline, and no or minimum functional impairment). Logistic regressions with inverse probability weighting accounting for demographic, behavioral, clinical covariates, and the complex sampling design were used to estimate odds ratios (ORs) and 95% confidence intervals (CIs) for the association between life‐course SEP and MCI.

**Results:**

9.8% of adults met criteria for MCI. High (vs. low) adult SEP was associated with lower odds of MCI (OR=0.78, 95% CI=0.60, 1.00). Having a stable high (vs. stable low) SEP over the life‐course (OR=0.68, 95% CI:=0.47, 0.98) was associated with lower odds of MCI. Childhood SEP (OR=0.87, 95% CI=0.65, 1.17) and socioeconomic mobility (OR for upward=0.79, 95% CI: 0.58, 1.08; and downward: OR=1.12, 95% CI: 0.67, 1.87) were not significantly associated with MCI.

**Conclusions:**

A Higher and stable life‐course SEP is associated with better cognitive aging in middle‐aged and older Hispanic/Latino adults, a population that is expected to have the largest increase in ADRD cases in the next four decades.

